# Acute Kidney Injury after Major Abdominal Surgery: A Retrospective Cohort Analysis

**DOI:** 10.1155/2014/132175

**Published:** 2014-02-24

**Authors:** Catarina Teixeira, Rosário Rosa, Natacha Rodrigues, Inês Mendes, Lígia Peixoto, Sofia Dias, Maria João Melo, Marta Pereira, Henrique Bicha Castelo, José António Lopes

**Affiliations:** ^1^Service of Nephrology and Renal Transplantation, Department of Medicine, Centro Hospitalar Lisboa Norte, EPE, Avenue Prof. Egas Moniz, 1649-035 Lisboa, Portugal; ^2^Service of Surgery II, Department of Surgery, Centro Hospitalar Lisboa Norte, EPE, Avenue Prof. Egas Moniz, 1649-035 Lisboa, Portugal

## Abstract

*Background*. We analyzed the incidence, risk factors, and prognosis of acute kidney injury (AKI) in a cohort of patients undergoing major abdominal surgery. * Methods*. A total of 450 patients were retrospectively studied. AKI was defined by an increase in serum creatinine (SCr) ≥ 0.3 mg/dl or by an increase in SCr ≥ 50% and/or by a decrease in urine output to 0.5 ml/kg/hour for 6 hours, in the first 48 hours after surgery. Logistic regression method was used to determine predictors of AKI and in-hospital mortality. A two-tailed *P* value <0.05 was considered significant. * Results*. One hundred one patients (22.4%) had postoperative AKI. Age (adjusted odds ratio (OR) 1.02, 95% confidence interval (CI) 1.01–1.05), nonrenal Revised Cardiac Risk Index score (adjusted OR 1.9, 95% CI 1.3–3.1, *P* = 0.003), intraoperative erythrocytes transfusions (adjusted OR 2.2, 95% CI 1.4–3.5, *P <*.0001), and nonrenal Simplified Acute Physiology Score II (adjusted OR 1.03, 95% CI 1.01–1.06, *P* = 0.0191) were associated with postoperative AKI. AKI was associated with increased in-hospital mortality (20.8% versus 2.3%, *P <*.0001; unadjusted OR 11.2, 95% CI 4.8–26.2, *P <*.0001; adjusted OR 3.7, 95% CI 1.2–11.7, *P* = 0.024). * Conclusion*. AKI was common in patients undergoing major abdominal surgery and was associated with in-hospital mortality.

## 1. Introduction

Acute kidney injury (AKI) occurs commonly in hospitalized patients and it has a detrimental impact on patient outcome. In fact, AKI is associated with increased costs, length of hospital stay and mortality [1–4].

The clinical characteristics and the impact of AKI in cardiac surgery has been extensively studied [5–9], and most of the published data regarding AKI in noncardiac surgery population are limited to high-risk aortic procedures [10–15]. Only few studies addressed AKI after noncardiac and nonvascular surgery [16–20] which has a distinct pathophysiology from cardiac and vascular surgery. As a result, it is inappropriate to assume that the risk factors for AKI after noncardiac and nonvascular surgery are the same as those after cardiac and vascular surgery. The purpose of this study was therefore to evaluate the incidence, risk factors, and outcome of AKI in the first 48 hours after surgery in a cohort of patients undergoing major nonvascular abdominal surgery.

## 2. Methods 

Patients aged 18 years or more who underwent scheduled or urgent major nonvascular abdominal surgery admitted to the Post-Anesthesia Care Unit (PACU) of the Service of Surgery II of the Centro Hospitalar Lisboa Norte, EPE (Lisbon, Portugal), between January 2010 and February 2011 were eligible for the study. Centro Hospitalar Lisboa Norte, EPE, comprises two tertiary and teaching hospitals (Hospital de Santa Maria and Hospital de Pulido Valente) providing medical assistance to an area with almost 3.000.000 inhabitants. The PACU is an eight bed unit in which surgical patients are admitted and closely monitored in the postoperative period, being managed by a dedicated intensivist.

Major abdominal surgery was considered whenever intraperitoneal approach under general anesthesia was performed and the predictable length of stay for patients in a given diagnosis-related group exceeded two days [21–23]. Exclusion criteria were chronic kidney disease on renal replacement therapy, requirement for renal replacement therapy in the week before surgery, less than 48 hours of hospital stay, and/or less than two determinations of serum creatinine (SCr).

For patients undergoing more than one surgery within the same hospitalization only the first surgery was considered for analysis.

Since this was a retrospective and observational study that did not evaluate a specific therapeutic or prophylactic intervention, study approval was waived by the Ethical Committee of our Hospital according to Institutional guidelines.

### 2.1. Variables

Manual patient medical charts and electronic hospital database were reviewed by eight investigators (CT, RR, NR, IM, LP, SD, MJM, and MP) to study demographic data (age, gender, and ethnicity), preoperative patient characteristics [scheduled versus urgent surgery, comorbidity, namely, diabetes mellitus, hypertension, ischemic heart disease, congestive heart failure, cerebrovascular disease, chronic obstructive pulmonary disease (COPD), cirrhosis, solid malignancy, hematological malignancy, the American Society of Anesthesiologists (ASA) score, hemoglobin, and SCr], surgical procedure, intraoperative patient characteristics (duration of anesthesia, blood pressure, fluids, and vasoactive drugs), Simplified Acute Physiology Score version II (SAPS II), postoperative complications (hemorrhage, anastomotic leak, surgical site infection, need for mechanical ventilation, and AKI), fluid balance, need for renal replacement therapy, intensive care unit (ICU) admission, and in-hospital mortality.

### 2.2. Definitions

Acute kidney injury was defined and staged by to the recently proposed Kidney Disease Improving Global Outcomes (KDIGO) classification [24]. Accordingly, AKI was defined by an increase in absolute SCr of at least 0.3 mg/dL or by a percentual increase in SCr equal to or higher than 50% (1.5X baseline value) and/or by a decrease in urine output (UO) (documented oliguria lower than 0.5 mL/kg/hour for more than 6 hours) [24] and staged as follows: stage 1, if there was an increase in absolute SCr of at least 0.3 mg/dL or a percentual increase in SCr 1.5 to 2X baseline value and/or a decrease in UO (documented oliguria lower than 0.5 mL/kg/hour for more than 6 hours); stage 2, if there was a percentual increase in SCr 2 to 3X baseline value and/or a decrease in UO (documented oliguria lower than 0.5 mL/kg/hour for more than 12 hours); and stage 3, if there was a percentual increase in SCr equal to or higher than 3X baseline value and/or a decrease in UO (documented oliguria lower than 0.3 mL/kg/hour for more than 24 hours or anuria for more than 12 hours) or in patients with baseline SCr > 4.0 mg/dL if the patient first achieved the Cr based change specified in the definition (either ≥ 0.3 mg/dL or an increase of ≥ 1.5X baseline value) or if patients required renal replacement therapy independently of the stage (defined by SCr and/or UO) they were in at the moment they initiated renal replacement therapy. In order to relate AKI with surgical procedure itself, thus eliminating the potential influence of other factors arising in the postoperative period, AKI was only considered in the first 48 hours after surgery. Preoperative SCr was considered baseline SCr.

Diabetes mellitus was diagnosed according to the World Health Organization criteria [25] and hypertension was diagnosed based on the seventh report of the Joint National Committee (JNC 7) [26].

Cerebrovascular disease was defined as history of transient ischemic attack or history of cerebrovascular accident and COPD included emphysema and chronic bronchitis.

The ASA score was used to evaluate preoperative patients' physical status [27].

The Revised Cardiac Risk Index (RCRI) score [22], assigning one point for each of the following risk factors: high-risk surgery, ischemic heart disease, congestive heart failure, cerebrovascular disease, diabetes mellitus requiring insulin therapy, and preoperative SCr higher than 2.0 mg/dL, was calculated to characterize the patients at risk to develop postoperative complications. As intraperitoneal procedure was performed, surgery was considered as high-risk in all cases [22].

Intraoperative systolic and diastolic blood pressure (SBP and DBP, resp.) were recorded every 5 minutes and intraoperative mean arterial pressure (MAP) was calculated as [(2 × DBP) + SBP]/3. When blood pressure was measured both invasively and noninvasively, invasive measurements were used for the current analysis. Intraoperative hypotension (IOH) was defined as intraoperative mean arterial pressure (MAP) <65 mmHg. The number of IOH episodes was registered.

The hemoglobin level justifying erythrocytes transfusion was variable and at physician discretion in patients with active hemorrhage and/or in hemodynamically unstable patients. Conversely, erythrocytes transfusion was performed if hemoglobin level was 7-8 g/dL (alternatively 9-10 g/dL in aged patients and/or in patients with acute coronary syndrome) in hemodynamically stable euvolemic patients [28, 29].

The SAPS II was used to evaluate illness severity and was calculated based on the worst variables recorded during the first 24 hours of PACU admission [30].

### 2.3. Statistical Analysis

Continuous variables are expressed as mean (standard deviation) and categorical variables as percentage of number of cases. Comparisons between patients with and without AKI were performed using the Student's *t*-test and the *χ* ^2^-test, respectively, for continuous and categorical variables. Logistic regression method was used to determine independent predictors of AKI and in-hospital mortality. Risk factors for AKI (age, gender, race, malignancy, ASA, nonrenal RCRI, preoperative hemoglobin, preoperative SCr, type of surgery, duration of surgery, IOH episodes, intraoperative fluids, intraoperative vasoactive drugs, and nonrenal SAPS II) and risk factors of in-hospital mortality (age, gender, race, malignancy, ASA, nonrenal RCRI, preoperative hemoglobin, preoperative SCr, type of surgery, nonrenal SAPS II, and fluid balance) were initially assessed with univariate analysis, and variables that were statistically significant (*P* < 0.05) in the univariate analysis were then included in the multivariate analysis with forward conditional elimination of data. Continuous variables were entered in the model as continuous variables and categorical variables as categorical ones. As RCRI includes one point to SCr and as SAPS II includes points for kidney insufficiency, the nonrenal RCRI and the nonrenal SAPS II were chosen as covariates to control for multicollinearity with SCr and AKI, respectively. Data are presented as odds ratios (ORs) with 95% confidence intervals (CIs). A two-tailed *P* value <0.05 was considered significant. Analysis was performed with the statistical software package SPSS 21.0 for Windows (Produtos e Serviços de Estatísticas, Lisboa, Portugal).

## 3. Results

During the study period, 492 patients underwent nonvascular major abdominal surgery in the Service of Surgery II of our Hospital; 10 of them were chronic kidney disease patients on dialysis and were not included. None of the patients had received a renal transplant or required renal replacement therapy in the week before surgery. From the 482 remaining patients, 32 patients were hospitalized less than 48 hours and/or had less than two SCr determinations within the hospitalization and were excluded from the analysis. Therefore, in this study, we focused on 450 patients and analyzed them as a cohort. Patient characteristics are described in Table 1.

### 3.1. Incidence and Risk Factors of AKI

One-hundred one patients (22.4%) developed AKI in the first 48 hours after surgery, as follows: 64 patients (63.4%) were at stage 1, 20 patients (19.8%) were at stage 2, and 17 patients were at stage 3 (16.8%). Patients with postoperative AKI were older (*P* < 0.0001) and were more likely to have preexisting ischemic heart disease (*P* = 0.001), preexisting congestive heart failure (*P* = 0.008), preexisting cerebrovascular disease (*P* < 0.0001), preexisting COPD (*P* < 0.0001), and solid malignancies (*P* = 0.005), to be ASA IV/V (*P* = 0.009), to have higher RCRI score (*P* < 0.0001) and nonrenal RCRI score (*P* < 0.0001). Additionally, patients with AKI were more likely to have lower preoperative hemoglobin levels (*P* < 0.0001) and higher preoperative SCr (*P* < 0.0001). Colorectal surgery (*P* = 0.006), longer duration of anesthesia (*P* = 0.021), intraoperative hypotension episodes (*P* < 0.0001), intraoperative colloids, and crystalloids as compared with crystalloids without colloids (*P* < 0.0001), as well as the amount of colloids used (*P* < 0.0001), intraoperative erythrocytes transfusions (*P* < 0.0001), and intraoperative vasoactive drugs (*P* = 0.037) were associated with increased incidence of postoperative AKI. Illness severity as evaluated by SAPS II (*P* < 0.0001) and nonrenal SAPS II (*P* < 0.0001) were also higher in patients with postoperative AKI than in those patients who did not have AKI (Table 2). Age (adjusted OR 1.02 per year, 95% CI 1.01–1.05 per year), nonrenal RCRI (adjusted OR 1.9 per point, 95% CI 1.3–3.1, *P* = 0.003), intraoperative erythrocytes transfusions (adjusted OR 2.2 per unit, 95% CI 1.4–3.5, *P* < 0.0001), and nonrenal SAPS II (adjusted OR 1.03 per point, 95% CI 1.01–1.06, *P* = 0.0191) were independently associated with postoperative AKI (Table 3).

### 3.2. Outcome

Ten out of the 101 patients with AKI (9.9%) received renal replacement therapy (eight patients were treated with continuous venovenous hemodiafiltration and two patients with intermittent hemodialysis). Patients with postoperative AKI were more likely to develop postoperative complications such as hemorrhage (*P* < 0.0001) and anastomotic leak (*P* = 0.005), were more likely to be transferred to ICU (*P* = 0.021), and had lengthened PACU (*P* < 0.0001) and hospital stay (*P* = 0.004) (Table 4).

Patients with postoperative AKI had higher in-hospital mortality than those patients who did not develop AKI (20.8 versus 2.3%, *P* < 0.0001; unadjusted OR 11.2, 95% CI 4.8–26.2, *P* < 0.001). After adjusting for other covariates, AKI was independently associated with increased in-hospital mortality (adjusted OR 3.7, 95% CI 1.2–11.7, *P* = 0.024) (Table 5). Moderate-to-severe AKI (stages 2 and 3) (in-hospital mortality 62%; unadjusted OR 36.6, 95% CI 15.1–89, *P* < 0.0001; adjusted OR 27.8, 95% CI 6.2–125, *P* < 0.0001) was associated with increased mortality, while mild AKI (stage 1) (in-hospital mortality 4.6%, unadjusted OR 0.7, 95% CI 0.2–2.3, *P* = 0.519) was not. Causes of death in patients with postoperative AKI were sepsis in 10 patients (47.6%), cardiovascular disease in 4 patients (19%), and respiratory failure in 4 patients (19%) and it was unknown in 3 patients (14.3%); sepsis (4 patients, 50%), cardiovascular disease (2 patients, 25%), respiratory failure (one patient, 12.5%), and unknown cause (one patient, 12.5%) were the causes of death in patients who did not have postoperative AKI.

## 4. Discussion

We analyzed retrospectively the incidence, predictors, and impact on outcome of AKI in a cohort of 450 patients undergoing major nonvascular abdominal surgery.

The incidence of AKI (22.4%) was similar to that reported for cardiac surgery patients (26 to 30%) [31, 32] and higher than that described for major noncardiac surgery population (7.5%) [17]. Causey and colleagues reported an incidence of postoperative AKI of 11.8% in patients submitted to colorectal surgery. In this study, AKI was defined as an increase in SCr ≥ 50% and not considered an increase in SCr ≥ 0.3 mg/dL within a 48-hour timeframe and UO variations [18]. A large multicentric study performed in mixed cardiac and noncardiac surgery population described a lower incidence of postoperative AKI (1.1%). Exclusion of patients with preoperative SCr > 1.2 mg/dL or preoperative glomerular filtration rate (GFR) < 60 mL/min and a less broad definition of AKI (a rise in SCr level to 2X the baseline value or a 50% reduction in the GFR, not considering UO changes) could be at least in part responsible for this observation [33].

There have been a variety of predictive models developed to stratify risk in patients undergoing cardiac surgery [34–36] and major noncardiac surgery [16]. Similarly to Abelha and colleagues [17] we found an association between the RCRI score [22] with postoperative AKI in patients undergoing major noncardiac surgery. It should be remembered that RCRI score was developed for the prediction of cardiac risk based on six independent prognostic factors: high-risk surgery, ischemic heart disease, congestive heart disease, history of cerebrovascular disease, insulin therapy for diabetes, and preoperative SCr higher than 2.0 mg/dL. An ischemic insult to a more sensitive kidney could therefore explain why RCRI can predict postoperative AKI.

Although patients undergoing urgent surgery were more likely to have preexisting congestive heart failure and solid malignancies, to be ASA IV/V and to have higher preoperative SCr and higher nonrenal SAPS II, as well as to undergo colorectal surgery, to have longer duration of anesthesia and intraoperative hypotension episodes, and to receive intraoperative colloids and intraoperative erythrocytes transfusions (data not shown), characteristics also found in AKI patients, urgent surgery was not associated with increased risk of postoperative AKI. Unfortunately, we are not able to explain this phenomenon which has also been recently described in a large mixed cardiac and noncardiac surgery population [33].

Laparoscopic procedures require the creation of a pneumoperitoneum by insufflation of CO2. There appears to be sufficient evidence to conclude that both renal function and renal blood flow are decreased during pneumoperitoneum mostly due to the increased intraabdominal pressure and the associated hormonal modifications [37]. Nonetheless, we found a similar incidence of AKI between patients undergoing laparoscopy and patients submitted to laparotomy. Similar preoperative renal function (data not shown), an adequate intraoperative hydration status, and increased abdominal pressure usually monitored and limited to 12 mmHg could explain at least in part this finding.

Intraoperative erythrocytes transfusions were a risk factor for postoperative AKI independently of the preoperative hemoglobin level. Although the intended therapeutic effect of erythrocytes transfusion is to improve organ function by increasing tissue oxygen delivery, there is increasing evidence that transfused erythrocytes may actually contribute to organ injury in susceptible patients, particularly in patients undergoing cardiac surgery in whom perioperative transfusion is a risk factor for renal dysfunction and is associated with worse outcomes [38–40]. Transfused stored erythrocytes may impair tissue oxygen delivery, promote a proinflammatory state, exacerbate tissue oxidative stress, and activate leukocytes and the coagulation cascade [41–43].

Patients with AKI had an in-hospital mortality of 20.8% which was slightly lower than that reported in a mixed noncardiac surgery population (27%) [17] and higher than that described in patients undergoing cardiac surgery (5 to 9%) [31, 32, 44, 45]. In a large mixed cardiac and noncardiac surgery population, 30-day and 3-month mortality of patients with AKI were 36% and 48%, respectively [33].

Postoperative AKI was independently associated with increased in-hospital mortality (patients with AKI were 3.7 times more likely to die) and there was a relationship between more severe AKI and increased in-hospital mortality. Despite the fact that AKI has emerged as an independent risk factor of in-hospital mortality, it is not clinically unequivocal if AKI may be per se an aspect of worse clinical condition/surgery (our findings are in line with previous studies [1–3] which have shown that AKI predictors and AKI outcomes are closely related) or if AKI itself independently contributes to increased mortality. The mechanism by which AKI itself could contribute to increased mortality remains not completely understood but volume overload, coagulation abnormalities, an increased incidence of sepsis with multiorgan failure, and cytokine or immune-mediated major organ dysfunction are possible explanations [46, 47]. In the present study, causes of death were similar between patients with postoperative AKI and patients who did not have AKI in the postoperative period. Therefore, further studies are needed to ascertain the exact mechanisms by which AKI could confer an increased mortality in patients undergoing major nonvascular abdominal surgery.

This study has some important limitations. First, the single-centre nature of the study largely limits its generalizability, and its retrospective design with a relatively small cohort of patients did not allow the evaluation of other confounders with prognostic importance. Second, we were not able to conclude about the exact mechanisms contributing to increased mortality in postoperative AKI patients submitted to major nonvascular abdominal surgery.

Our study has, however, some important virtues. First, it characterizes, for the first time, the incidence, risk factors, and impact on outcome of AKI (defined according to standardized definition using simultaneously SCr and UO criteria) in major nonvascular abdominal surgery. Second, despite its retrospective design, studied variables were routinely registered in daily practice. Third, many covariates with impact on AKI development and outcome were analyzed. Finally, causes of death were attained.

## Figures and Tables

**Table 1:** Preoperative characteristics, type of surgery, intraoperative characteristics, postoperative complications, length of stay, and mortality of patients (*N* = 450) undergoing major nonvascular abdominal surgery.

Variable	
Preoperative characteristics	
Age (years)	62 (16)
Male *N* (%)	227 (50.4)
Caucasian *N* (%)	431 (95.8)
Diabetes mellitus *N* (%)	84 (18.7)
Hypertension *N* (%)	225 (50)
Ischemic heart disease *N* (%)	54 (12)
Congestive heart failure *N* (%)	25 (5.6)
Cerebrovascular disease *N* (%)	29 (6.4)
COPD *N* (%)	25 (5.6)
Cirrhosis *N* (%)	9 (2)
Solid malignancy *N* (%)	190 (42.2)
Hematological malignancy *N* (%)	8 (1.8)
ASA IV/V *N* (%)	48 (10.7)
RCRI score	1.3 (0.6)
Nonrenal RCRI	1.2 (0.5)
Preoperative hemoglobin (g/dL)	11.1 (1.7)
Preoperative SCr (mg/dL)	1 (0.5)
Urgency surgery *N* (%)	101 (22.4)
Type of surgery	
Laparoscopy *N* (%)	55 (12.2)
Laparotomy *N* (%)	390 (86.7)
Laparoscopy converted to laparotomy *N* (%)	5 (1.1)
Colorectal *N* (%)	222 (49.3)
Gastric *N* (%)	85 (18.9)
Hepatobiliary and pancreatic^1^ *N* (%)	65 (14.4)
Small bowel *N* (%)	19 (4.2)
Esophageal *N* (%)	11 (2.4)
Other surgery *N* (%)	48 (10.7)
Intraoperative characteristics	
Duration of anestesia (min.)	223 (100)
IOH (number of records)	1 (1.8)
Crystalloids without colloids *N* (%)	175 (38.9)
Crystalloids and colloids *N* (%)	275 (61.1)
Erythrocytes (units)	0.5 (0.7)
Vasoactive drugs use *N* (%)	80 (17.7)
Postoperative complications	
Hemorrhage *N* (%)	45 (10)
Anastomotic leak *N* (%)	21 (4.7)
Surgical site infection *N* (%)	71 (15.8)
Need for MV *N* (%)	11 (2.4)
Admission to ICU *N* (%)	13 (2.9)
SAPS II	23 (17.4)
Nonrenal SAPS II	20.8 (15.4)
Length of PACU stay (days)	4.4 (8.6)
Length of hospital stay (days)	12 (12.6)
PACU mortality *N* (%)	20 (4.4)
In-hospital mortality *N* (%)	29 (6.4)

COPD: chronic obstructive pulmonary disease. ASA: American Society of Anesthesiologists. RCRI: Revised Cardiac Risk Index. SCr: serum creatinine. IOH: intraoperative hypotension. MV: mechanical ventilation. ICU: intensive care unit. SAPS II: Simplified Acute Physiology Score, version II. PACU: Post-Anaesthesia Care Unit. ^1^Hepatobiliary and pancreatic surgery includes gallbladder surgery (*N* = 40), hepatic surgery (*N* = 15), pancreatic surgery (*N* = 6), and bile ducts surgery (*N* = 4).

**Table 2:** Characteristics of patients with postoperative AKI (*N* = 101) and with no postoperative AKI (*N* = 349).

Variable	No AKI (*N* = 349)	AKI (*N* = 101)	*P*
Preoperative characteristics			
Age (years)	60.6 (15.7)	71.1 (13.2)	<0.0001
Male *N* (%)	168 (48.1)	59 (58.4)	0.069
Caucasian *N* (%)	335 (96)	96 (95)	0.679
Diabetes mellitus *N* (%)	62 (17.8)	22 (21.8)	0.362
Hypertension *N* (%)	168 (48.1)	57 (56.4)	0.142
Ischemic heart disease *N* (%)	32 (9.2)	22 (21.8)	0.001
Congestive heart failure *N* (%)	14 (4)	11 (10.9)	0.008
Cerebrovascular disease *N* (%)	13 (3.7)	16 (15.8)	<0.0001
COPD *N* (%)	13 (3.7)	12 (11.9)	<0.0001
Cirrhosis *N* (%)	7 (2)	2 (1.9)	0.987
Solid malignancy *N* (%)	135 (38.7)	55 (54.5)	0.005
Hematological malignancy *N* (%)	6 (1.7)	2 (1.9)	0.861
ASA IV/V *N* (%)	23 (6.6)	15 (14.9)	0.009
RCRI score	1.2 (0.5)	1.6 (0.8)	<0.0001
Nonrenal RCRI score	1.2 (0.4)	1.5 (0.8)	<0.0001
Preoperative hemoglobin (g/dL)	11.3 (1.7)	10.5 (1.6)	<0.0001
Preoperative SCr (mg/dL)	0.9 (0.4)	1.2 (0.7)	<0.0001
Urgency surgery *N* (%)	75 (21.5)	26 (25.7)	0.367
Type of surgery			
Laparoscopy *N* (%)	47 (13.5)	8 (7.9)	0.134
Laparotomy *N* (%)	297 (85.1)	93 (92)	0.07
Colorectal *N* (%)	160 (45.8)	62 (61.4)	0.006
Gastric *N* (%)	71 (20.3)	14 (13.9)	0.143
Hepatobiliary and pancreatic^1^ *N* (%)	55 (15.8)	10 (9.9)	0.140
Small bowel *N* (%)	14 (4)	5 (4.9)	0.679
Esophageal *N* (%)	8 (2.3)	3 (2.9)	0.698
Other surgery *N* (%)	41 (11.7)	7 (6.9)	0.167
Intraoperative characteristics			
Duration of anestesia (min.)	218 (92)	244 (121)	0.021
IOH (number of records)	0.9 (1.7)	1.6 (2.1)	<0.0001
Crystalloids without colloids *N* (%)	80 (29)	21 (12)	<0.0001
Amount of Crystalloids (L)	2.3 (1.3)	2.3 (1.3)	0.905
Crystalloids and colloids *N* (%)	21 (12)	80 (29)	<0.0001
Amount of Crystalloids (L)	2.2 (1.1)	2.5 (1.5)	0.054
Amount of colloids (L)	0.4 (0.3)	0.7 (0.4)	<0.0001
Erythrocytes (units)	0.4 (0.5)	1 (1)	<0.0001
Vasoactive drugs use *N* (%)	55 (15.8)	25 (24.8)	0.037
SAPS II	19.1 (10.9)	34.3 (21.3)	<0.0001
Nonrenal SAPS II	17.1 (9.6)	28.4 (17.6)	<0.0001
Fluid balance (liters)^2^	2.4 (2.2)	2.6 (2.4)	0.475

COPD: chronic obstructive pulmonary disease. ASA: American Society of Anesthesiologists. RCRI: Revised Cardiac Risk Index. SCr: serum creatinine. IOH: intraoperative hypotension. SAPS II: Simplified Acute Physiology Score, version II. ^1^Hepatobiliary and pancreatic surgery includes gallbladder surgery (*N* = 40), hepatic surgery (*N* = 15), pancreatic surgery (*N* = 6), and bile ducts surgery (*N* = 4). ^2^Fluid balance within the first two postoperative days.

**Table 3:** Univariate and multivariate analysis to determine risk factors of postoperative acute kidney injury.

Variable	Unadjusted OR (95% CI)	*P*	Adjusted OR (95% CI)	*P*
Preoperative characteristics				
Age (per year)	1.05 (1.04–1.07)	<0.0001	1.02 (1.01–1.05)	0.049
Male	1.5 (0.9–2.4)	0.07		
Caucasian	0.8 (0.3–2.3)	0.680		
Solid malignancy	1.9 (1.2–2.9)	0.005	1.4 (0.8–2.4)	0.274
ASA physical status IV/V	2.5 (1.2–4.9)	0.01	1.1 (0.4–2.5)	0.931
Nonrenal RCRI (per point)	2.9 (1.9–4.3)	<0.0001	1.9 (1.3–3.1)	0.003
Preoperative hemoglobin (per g/dL)	0.8 (0.7–0.9)	<0.0001	1 (0.8–1.2)	0.825
Preoperative SCr (per mg/dL increase)	2.3 (1.5–3.4)	<0.0001	1.2 (0.7–2)	0.411
Urgency surgery	1.3 (0.8–2.1)	0.368		
Type of surgery				
Laparoscopy versus laparotomy	0.6 (0.3–1.2)	0.139		
Colorectal	1.9 (1.2–2.9)	0.006	1.6 (0.9–2.7)	0.122
Gastric	0.6 (0.3–1.1)	0.630		
Hepatobiliary and pancreatic^1^	0.6 (0.3–1.2)	0.144		
Small bowel	1.2 (0.4–3.5)	0.680		
Esophageal	1.3 (0.3–5)	0.698		
Other surgery	0.6 (0.2–1.3)	0.559		
Intraoperative characteristics				
Duration of anesthesia (per 10 min.)	1.02 (1–1.05)	0.024	1.01 (0.9–1.1)	0.208
IOH (per record)	1.2 (1.1–1.4)	0.001	1.1 (0.9–1.2)	0.437
Crystalloids with colloids versus crystalloids without colloids	3 (1.8–5)	<0.0001	1.6 (0.9–3)	0.143
Erythrocytes (per unit)	3.7 (2.4–5.8)	<0.0001	2.2 (1.4–3.5)	<0.0001
Vasoactive drugs use	1.8 (1.1–3)	0.039	1.1 (0.5–2)	0.939
Nonrenal SAPS II (per point)	1.07 (1.05–1.09)	<0.0001	1.03 (1.01–1.06)	0.019

OR: odds ratio. CI: confidence interval. COPD: ASA: American Society of Anesthesiologists. RCRI: Revised Cardiac Risk Index. SCr: serum creatinine. IOH: intraoperative hypotension. SAPS II: Simplified Acute Physiology Score, version II. ^1^Hepatobiliary and pancreatic surgery includes gallbladder surgery (*N* = 40), hepatic surgery (*N* = 15), pancreatic surgery (*N* = 6), and bile ducts surgery (*N* = 4).

**Table 4:** Postoperative complications, length of stay, and mortality.

Postoperative complications	No AKI (*N* = 349)	AKI (*N* = 101)	*P*
Hemorrhage *N* (%)	23 (6.6)	22 (21.8)	<0.0001
Anastomotic leak *N* (%)	11 (3.2)	10 (10)	0.005
Surgical site infection *N* (%)	53 (15.2)	18 (17.8)	0.521
Need for MV *N* (%)	6 (1.7)	5 (4.9)	0.064
Admission to ICU *N* (%)	6 (1.7)	6 (5.9)	0.021
Length of PACU stay (days)	3.6 (7.1)	6.9 (11.7)	<0.0001
Length of hospital stay (days)	11.1 (11.3)	15.2 (16.1)	0.004
PACU mortality *N* (%)	4 (1.1)	16 (15.8)	<0.0001
In-hospital mortality *N* (%)	8 (2.3)	21 (20.8)	<0.0001

AKI: acute kidney injury. PACU: Post-Anaesthesia Care Unit. MV: mechanical ventilation. ICU: intensive care unit.

**Table 5:** Univariate and multivariate analysis to determine predictors of in-hospital mortality.

Variable	Unadjusted OR (95% CI)	*P*	Adjusted OR (95% CI)	*P*
Preoperative characteristics				
Age (per year)	1.1 (1.06–1.2)	<0.0001	1.07 (1.02–1.1)	0.014
Male	1.1 (0.5–2.2)	0.887		
Caucasian	1.3 (1.2–9.7)	0.831		
Solid malignancy	1.5 (0.7–3.2)	0.287		
ASA physical status IV/V	15.4 (6.7–35.6)	<0.0001	9.1 (2.9–27.5)	<0.0001
Nonrenal RCRI (per point)	2.6 (1.5–4.3)	<0.0001	1.6 (0.8–3.2)	0.213
Preoperative hemoglobin (per g/dL)	0.63 (0.5–0.8)	<0.0001	0.9 (0.6–1.2)	0.501
Preoperative SCr (per mg/dL)	2.3 (1.3–3.9)	0.003	0.6 (0.3–1.4)	0.268
Urgency surgery	4.2 (1.9–8.9)	<0.0001	1.9 (0.6–6.3)	0.320
Type of surgery				
Colorectal	1.3 (0.6–2.7)	0.516		
Gastric	0.7 (0.2–1.9)	0.471		
Hepatobiliary and pancreatic^1^	0.2 (0.03–1.5)	0.116		
Small bowel	0.8 (0.1–6.2)	0.800		
Esophageal	3.4 (0.7–16.5)	0.130		
Other surgery	1.8 (0.7–5)	0.242		
Nonrenal SAPS II (per point)	1.13 (1.09–1.2)	<0.0001	1.08 (1.04–1.1)	<0.0001
Fluid balance (per liter)^2^	0.9 (0.8–1.1)	0.918		
Postoperative AKI	11.2 (4.8–26.2)	<0.0001	3.7 (1.2–11.7)	0.024

OR: odds ratio. CI: confidence interval. ASA: American Society of Anesthesiologists. RCRI: Revised Cardiac Risk Index. SCr: serum creatinine. SAPS II: Simplified Acute Physiology Score, version II. AKI: acute kidney injury. ^1^Hepatobiliary and pancreatic surgery includes gallbladder surgery (*N* = 40), hepatic surgery (*N* = 15), pancreatic surgery (*N* = 6), and bile ducts surgery (*N* = 4). ^2^Fluid balance within the first two postoperative days.
